# Oxaliplatin‐induced neuropathy after total neoadjuvant therapy for rectal cancer: Dose–response relationship and impact on quality of life

**DOI:** 10.1002/ijc.70396

**Published:** 2026-02-18

**Authors:** Georg W. Wurschi, Andreas Hinz, Melanie Schneider, Jan‐Niklas Becker, Bernd Frerker, Samuel M. Vorbach, Felix Ehret, Markus Diefenhardt, Fabian Schunn, Maria‐Elena von Gruben, Marcel Büttner, Elgin Hoffmann, Alexander Rühle, Josephine Beier, Simone Ferdinandus, Maike Trommer, Ezgi Ceren Sahin, Julian Hlouschek, Kynann Aninditha, Daphne Schepers von Ohlen, Justus Kaufmann, Alina Depardon, Hai Minh Ha, Christopher Kessler, Adrianna Cieslak, Simon Trommer, Alexander Fabian, Florian Rißner, Maximilian Römer, Klaus Pietschmann

**Affiliations:** ^1^ Department of Radiotherapy and Radiation Oncology Jena University Hospital Jena Germany; ^2^ Comprehensive Cancer Center Central Germany (CCCG), Partner Site Jena Jena Germany; ^3^ Department of Medical Psychology and Medical Sociology University Medical Center Leipzig Leipzig Germany; ^4^ Comprehensive Cancer Center Central Germany (CCCG), Partner Site Leipzig Leipzig Germany; ^5^ Department of Radiotherapy and Radiation Oncology, Faculty of Medicine University Hospital Carl Gustav Carus, Technische Universität Dresden Dresden Germany; ^6^ Department of Radiotherapy Hannover Medical School Hannover Germany; ^7^ Department of Radiotherapy and Radiation Oncology Rostock University Medical Center Rostock Germany; ^8^ Department of Radiation Oncology Medical University of Innsbruck Innsbruck Austria; ^9^ Charité ‐ Universitätsmedizin Berlin Corporate Member of Freie Universität Berlin and Humboldt‐Universität zu Berlin, Department of Radiation Oncology Berlin Germany; ^10^ German Cancer Consortium (DKTK), Partner Site Berlin, a partnership between DKFZ and Charité ‐ Universitätsmedizin Berlin Berlin Germany; ^11^ Department of Radiotherapy and Oncology, University Hospital Goethe University Frankfurt Frankfurt am Main Germany; ^12^ Frankfurt Cancer Institute (FCI) Frankfurt am Main Germany; ^13^ Department of Radiation Oncology University Hospital Heidelberg Heidelberg Germany; ^14^ Department of Radiotherapy and Radiation Oncology University Hospital Hamburg‐Eppendorf Hamburg Germany; ^15^ Department of Radiation Oncology University Hospital Tübingen Tübingen Germany; ^16^ German Cancer Consortium (DKTK), Partner Site Tübingen Tübingen Germany; ^17^ German Cancer Research Center (DKFZ) Heidelberg Germany; ^18^ Department of Radiation Oncology, Medical Center ‐ University of Freiburg, Faculty of Medicine, University of Freiburg, German Cancer Consortium (DKTK), Partner Site DKTK‐Freiburg Freiburg im Breisgau Germany; ^19^ Department of Radiation Oncology University Medical Center Leipzig Leipzig Germany; ^20^ Department of Radiation Oncology, Cyberknife and Radiotherapy Faculty of Medicine and University Hospital Cologne Köln Germany; ^21^ Center for Integrated Oncology Aachen Bonn Cologne Duesseldorf (CIO ABCD) Köln Germany; ^22^ Department I of Internal Medicine, Center of Integrated Oncology Aachen Bonn Cologne Düsseldorf University Hospital of Cologne Cologne Germany; ^23^ Department of Radiotherapy, West German Cancer Center University Hospital Essen Essen Germany; ^24^ Department of Radiooncology Klinikum Stuttgart Stuttgart Germany; ^25^ Department of Radiotherapy University Medical Center Schleswig‐Holstein/Lübeck Lübeck Germany; ^26^ Department of Radiooncology University Medical Center of the Johannes‐Gutenberg‐University Mainz Mainz Germany; ^27^ Department of Radiation Oncology Universitätsklinikum Erlangen, Friedrich‐Alexander‐Universität Erlangen‐Nürnberg Erlangen Germany; ^28^ Department of Radiation Oncology Otto von Guericke Universität Magdeburg Magdeburg Germany; ^29^ Department of Radiation Oncology Technical University of Munich (TUM), School of Medicine and Klinikum Rechts der Isar München Germany; ^30^ Department of Radiation Oncology University Medicine Mannheim, Medical Faculty Mannheim, Heidelberg University Mannheim Germany; ^31^ Center for Radiotherapy and Radiation Oncology Bremen Germany; ^32^ Department for Radiotherapy University Hospital Halle Halle (Saale) Germany; ^33^ Department of Radiation Oncology University Hospital Schleswig‐Holstein Campus Kiel Kiel Germany; ^34^ Center for Clinical Studies Jena University Hospital Jena Germany

**Keywords:** CIPN, oxaliplatin, polyneuropathy, rectal cancer, total neoadjuvant therapy

## Abstract

Oxaliplatin‐based regimens are increasingly used in total neoadjuvant therapy (TNT) for locally advanced rectal cancer, frequently causing dose‐limiting chemotherapy‐induced peripheral neuropathy (CIPN), whose extent and impact on health‐related quality of life (HrQoL) remain insufficiently characterized. The optimal duration and intensity of chemotherapy within TNT for tumor response and toxicity remain unclear. This retrospective multicenter study (DRKS00033000) assessed the dose–response relationship of CIPN with cumulative oxaliplatin dose (cOXAd) and its impact on HrQoL. A total of 227 patients (164 men) with a median age of 63 (Q1–Q3: 54–68) years, who underwent oxaliplatin‐based TNT between 2015 and 2024, were analyzed. HrQoL was assessed cross‐sectionally during follow‐up using the EORTC QLQ‐C30 and QLQ‐CIPN20 questionnaires. Multivariable logistic regression was applied to evaluate the relationship between cOXAd and CIPN grade ≥2, and known‐group comparisons examined differences in HrQoL scores. At follow‐up, 61 patients (26.9%) experienced CIPN grade ≥2 after a median cOXAd of 758.8 mg/m^2^. Higher cOXAd was associated with an increased likelihood of CIPN grade ≥2 (adjusted OR 1.004, 95% CI: 1.002–1.007), corresponding to a 7.8% higher probability per additional FOLFOX cycle (+85 mg/m^2^ cOXAd). Patients with CIPN grade ≥2 reported lower mean QLQ‐C30 Global Health Scores and Summary Scores (−13.0 and −12.4 points; Cohen's *d* = −0.59 and −0.68) and higher QLQ‐CIPN20 Summary Scores (+23.9 points; Cohen's *d* = 1.65) compared to those without CIPN. These findings indicate that CIPN is a common dose‐dependent toxicity of oxaliplatin‐based TNT, associated with considerably reduced HrQoL and markedly increased neuropathy‐related symptom burden at follow‐up.

## What's New?

Oxaliplatin serves a key role in the treatment of rectal cancer, though it is a significant cause of chemotherapy‐induced peripheral neuropathy. As total neoadjuvant therapy, in which all therapy is given before surgery, has become more common, understanding neuropathy has grown increasingly important. This study examined neuropathy and its relationship with oxaliplatin dose and quality of life in patients with advanced rectal cancer. Neuropathy was found to be frequent after treatment, becoming more likely with each additional chemotherapy cycle. Moreover, it was associated with significantly worse quality of life, highlighting the importance of balancing treatment effectiveness with long‐term patient well‐being.

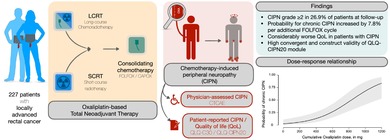

**Abbreviations**
95% CI: 95% confidence interval
ADL: activities of daily living
AUC: area under the curve
BSA: body surface area
CIPN: chemotherapy‐induced peripheral neuropathy
cOXAd: cumulative oxaliplatin dose, in mg/m^2^
CR: complete response
CRM: circumferential resection margin
CTCAE: Common Terminology Criteria for Adverse Events
DEGRO: Deutsche Gesellschaft für Radioonkologie (German Society for Radiation Oncology)
EMVI: extramural vascular invasion
ESMO: European Society for Medical Oncology
GHS: Global Health Scale (QLQ‐C30 subscale)
HrQoL: health‐related quality of life
KPS: Karnofsky Performance Status
LCRT: long‐course chemoradiotherapy
MRF: mesorectal fascia
NOM: non‐operative management
OR: odds ratio
PROM: patient‐reported outcome measurement
ROC: receiver‐operator characteristics
SCRT: short‐course radiotherapy
TNT: total neoadjuvant therapy
UICC: Union Internationale Contre le Cancer
VIF: variance influence factor

## 1 INTRODUCTION

Chemotherapy‐induced peripheral neuropathy (CIPN) is a common dose‐limiting toxicity of oxaliplatin.^1^ It usually occurs with increasing cumulative oxaliplatin doses (cOXAd) and may subsequently affect motor, sensory, and autonomic function in the long term, thereby reducing quality of life (QoL).^2^, ^3^ Typical symptoms include numbness, tingling, burning pain, reduced tactile and vibration sensation, impaired fine motor skills, gait instability, and, in the autonomic domain, dizziness or erectile dysfunction.^2^

Total neoadjuvant therapy (TNT) has emerged as a promising approach for the treatment of locally advanced rectal cancer in the presence of certain risk factors, improving both local control and reducing metastatic spread.^4^, ^5^, ^6^, ^7^ This treatment intensification further increases complete response (CR) rates to 25–65%,^8^, ^9^, ^10^ allowing non‐operative management (NOM) in an increasing number of patients.^11^, ^12^ Systemic treatment within TNT is most commonly FOLFOX‐ or FOLFOXIRI‐based, and in order to maximize tumor regression, long‐term regimens with 6–9 cycles are typically administered.^13^, ^14^ The resulting cOXAd frequently exceeds established thresholds for clinically relevant CIPN of >550 mg/m^2^,^2^ making it one of the dominant long‐term toxicities from the patient's perspective. Although the risk for CIPN may be heightened by certain factors such as diabetes, preexisting neuropathy, or smoking,^2^, ^15^ clinical risk factors in the specific setting of TNT, as well as its impact on health‐related quality of life (HrQoL), have not been adequately investigated. As TNT is increasingly applied in the context of intended NOM, additional toxicities must be weighed against the potential gains in function and QoL following rectum preservation. We therefore aimed to analyze the prevalence of CIPN, its dose–response relationship with oxaliplatin, and its impact on HrQoL in this multicenter cohort. The EORTC QLQ‐CIPN20 module, designed as a complementary module to the EORTC QLQ‐C30, has recently completed Phase III clinical validation, underscoring the need for real‐world application, as pursued in the present study.

## 2 MATERIALS AND METHODS

### 2.1 Study design and setting

We conducted a multicenter analysis within the “Young DEGRO” working group of the German Society for Radiation Oncology (DEGRO) at 23 hospitals in Germany and Austria,^16^ which are listed in detail in sect. 1 of Data S1, Supporting Information. Analyses were conducted in accordance with the STROBE statement.^17^

Eligible patients were diagnosed with localized rectal cancer (TNM classification: T2‐4N0‐2 M0/UICC stage II or III) between 2015 and 2024 and underwent neoadjuvant (chemo)radiotherapy followed by oxaliplatin‐based consolidation chemotherapy with curative intent. Radiotherapy was administered as hypofractionated short‐course radiotherapy (SCRT), i.e., 25 Gy/5 fractions, without concomitant chemotherapy, or long‐course chemoradiotherapy (LCRT) over 5–6 weeks, such as 50.4 Gy/28 fractions or 45 Gy/25 fractions with simultaneous or sequential boost and concomitant pyrimidine‐based chemotherapy. Treatment characteristics comprised the applied treatment schedule, including cOXAd, and the related clinician‐assessed “acute” toxicity. Follow‐up data, including clinician‐assessed “chronic” toxicity and tumor control, were collected during routine oncological follow‐up visits in accordance with institutional standards. The EORTC QLQ‐C30 and QLQ‐CIPN20 questionnaires were administered after obtaining the patients' consent during these follow‐up visits in a cross‐sectional design.

### 2.2 Questionnaires

Due to local regulatory and data protection requirements, questionnaires were available from 10 centers only. The QLQ‐C30 includes 30 questions on 4‐point or 7‐point multi‐item scales, incorporating five functioning scales (Physical, Role, Cognitive, Emotional, and Social), three symptom scales (Fatigue, Pain, Nausea and Vomiting), Global Health Status and additional single items assessing commonly reported symptoms (dyspnea, loss of appetite, insomnia, constipation and diarrhea, financial impact of the disease). The QLQ‐CIPN20 questionnaire contains 20 items, rating patients' symptoms on a 4‐point multi‐item scale.^18^ Item 19 rates difficulties regarding the use of pedals/driving a car, and item 20 rates male impotence. Subscales (range, 0–100) were calculated for both questionnaires (from the corresponding single item values) according to the EORTC manuals.^19^ Higher scores on functioning scales represent better function, whereas higher symptom scores indicate a high level of symptoms. QLQ‐C30 subscales were summarized in the established Summary Score^20^ to ease comparisons. The QLQ‐CIPN20 items were summarized to a Summary Score (items 1–19) as well as to Sensory, Motor, and Autonomic subscales.^18^, ^21^

### 2.3 Endpoints and definitions

The primary endpoint of this analysis is clinician‐assessed, “relevant,” i.e., CTCAE grade ≥2, CIPN at follow‐up. Secondary endpoints comprised patient‐reported HrQoL scores. Given the large number of single‐item scales, explorative testing was limited to the most relevant multi‐item scales reflecting ClPN, i.e., the QLQ‐CIPN20 “Summary Score” and the respective subscales, as well as general HrQoL, assessed per QLQ‐C30 “Summary Score” and QLQ‐C30 “Global Health Scale (GHS),” and the QLQ‐C30 subscale “Pain.” Within the prospective study protocol, clinical features, including demographic factors, such as age, sex, presence of diabetes, the Karnofsky performance status (KPS), or cumulative oxaliplatin dose (cOXAd, in mg/m^2^), were prespecified as factors for further explorative comparison.

Clinician‐assessed toxicity was graded according to the established Common Terminology Criteria for Adverse Events (CTCAE) v5.0 (0–5). Toxicities of grade ≥2 were considered “relevant,” and those of grade ≥3 were considered “severe.” Acute toxicity was defined as the highest grade observed per category during TNT, whereas chronic toxicity was assessed at follow‐up.

cOXAd was defined as the total oxaliplatin dose (in mg/m^2^), adjusted for body surface area (BSA) that was administered during both concomitant and consolidation phases, taking into account any dose reductions.

### 2.4 Statistical analysis

All analyses are of an exploratory nature. Therefore, no explicit sample size calculation was performed. Categorical variables were reported descriptively with absolute and relative frequencies (%); continuous variables with mean (±standard deviation, SD) and median (first and third quartile, Q1–Q3).

A logistic regression model was used to examine the association between chronic CIPN and clinical factors, with a particular focus on cOXAd (see sect. 2 of Data S1 for model specifications). Multicollinearity diagnostics were performed, and model performance was evaluated using the area under the receiver operating characteristic curve (AUC) and the Brier score.^22^ Adjusted odds ratios (OR) with 95% confidence intervals (95% CI) were reported. The optimal cut‐off for predicting chronic CIPN was determined using Youden's Index.^23^

Spearman's correlation was calculated for the evaluation of continuous variables, whereas known group comparisons were performed with Mann–Whitney *U* tests. Following common practice,^24^ these comparisons were limited to the QLQ‐C30 GHS and the QLQ‐C30 Summary Score^20^ of the QLQ‐C30 questionnaire. The internal consistency of QLQ‐CIPN20 Summary scales, as well as Sensory, Motor, and Autonomic subscales, was assessed using Cronbach's *α* and McDonald's *ω* prior to the application as these are not officially validated yet. Note that, according to the questionnaire manual, the Autonomic subscale comprised only two items and should be interpreted with caution. Known‐group comparisons were conducted for relevant parameters, and differences in means were reported together with corresponding effect sizes (Cohen's *d*). Only differences considered clinically relevant (>10 points^25^) were evaluated.

All 95% CI were obtained via nonparametric bootstrapping with 1000 resamples using the percentile method. A two‐sided significance level of *p* < .05 was applied for all statistical tests. Additionally, Holm–Bonferroni adjusted *p*‐values are provided for sensitivity analysis in multiple comparisons of subgroups. In descriptive analyses, missing values were excluded using pairwise deletion, resulting in different numbers of included cases per variable. Logistic regression models were restricted to complete cases. All analyses were conducted with SPSS 29.0 (IBM SPSS Statistics, Armonk, NY) as well as JASP v0.95.1 (JASP Team, 2025,^26^). Excel v16.96 (Microsoft Inc., Redmond, WA) and Keynote v14.4 (Apple Inc., Cupertino, CA) were used for further visualization.

## 3 RESULTS

### 3.1 Patient characteristics and treatments

We included 227 patients with a median age at diagnosis of 63 (Q1–Q3: 54–68) years. Among them, 164 (72.2%) were male. Most patients were in good general condition (median KPS of 90%) and, among them, 27 (11.9%) were diagnosed with diabetes prior to treatment. One‐hundred thirty‐one patients (57.7%) received concomitant CRT, and a median of 8 (Q1–Q3: 6–9) cycles of consolidation chemotherapy were administered. FOLFOX (149 patients/67.7%) and CAPOX (64 patients/29.1%) were the most commonly applied schedules. A median cOXAd of 658.8 (Q1–Q3: 500.0–765.0) mg/m^2^ was administered. The patient and treatment characteristics are summarized in Table 1.

**TABLE 1:** (A) Categorical variables are presented as absolute (*n*) and relative (%) frequencies for patient and treatment characteristics. (B) Continuous variables are presented as mean ± standard deviation (SD) and median with first (Q1) and third (Q3) quartiles for patient and treatment characteristics.

(A) Categorical variables	*n*	%
Sex (*N* = 227)		
Female (f)	63	27.8
Male (m)	164	72.2
Diabetes (*N* = 226)		
No	199	88.1
Yes	27	11.9
TNM T stage (*N* = 227)		
T1	3	1.3
T2	10	4.4
T3 a/b	107	47.1
T3 c/d	60	26.4
T4	47	20.7
TNM N stage (*N* = 227)		
N0	26	11.5
N1	67	29.5
N2	96	42.3
N+	36	15.9
Nx	2	0.9
TNM M stage (*N* = 227)		
M0	227	100.0
Grading pre‐treatment (*N* = 212)		
G1	10	4.7
G2	193	91.0
G3	9	4.2
Tumor localization (*N* = 227)		
0–6 cm from anal verge	123	54.2
6–12 cm from anal verge	100	44.1
>12 cm from anal verge	4	1.8
Involvement of lateral pelvic nodes (*N* = 217)		
No	170	78.3
Yes	47	21.7
EMVI positive tumor (*N* = 190)		
EMVI −	135	71.1
EMVI +	55	28.9
Involvement of CRM (*N* = 214)		
CRM involved (<1 mm)	97	45.3
CRM threatened (1–2 mm)	23	10.7
CRM clear (>2 mm)	94	43.9
ESMO tumor risk classification (*N* = 216)		
Early (good)	10	4.6
Intermediate	43	19.9
Bad	45	20.8
Advanced (ugly)	118	54.6
Fractionation schedule (*N* = 227)		
25.0 (5.0) Gy	96	42.3
50.4 (1.8) Gy or 50.0 (2.0) Gy	99	43.6
Boost concept (45.0 Gy + Boost)	25	11.0
Boost concept (50.4 Gy + Boost)	6	2.6
Other^a^	1	0.4
Chemoradiotherapy (*N* = 227)		
No (SCRT)	96	42.3
Yes (LCRT)	131	57.7
Concomitant CRT protocol (*N* = 131)^b^		
5‐FU continuous infusion (225 mg/sqm/d, continuous), e.g., OPRA protocol	2	1.5
5‐FU per ARO‐94 study (1000 mg/sqm/d, week 1 + 5)	20	15.3
5‐FU (250 mg/sqm/d) + oxaliplatin (50 mg/sqm), week 1, 2 + 4, 5, per ARO‐04 study	69	52.7
Capecitabine mono (1650 mg/sqm/d, daily), e.g., OPRA protocol	35	26.7
Other^c^	5	3.8
Not applicable (SCRT)^b^	96	
Sequential chemotherapy protocol (*N* = 220)		
FOLFOX	149	67.7
CAPOX	64	29.1
FOLFIRINOX	1	0.5
Other protocols^d^	6	2.7
Preexisting polyneuropathy prior to treatment (*N* = 225)		
No	222	98.7
Yes	3	1.3

(B) Continuous variables								
	*N*	Mean	SD	Median	Q1	Q3	Minimum	Maximum
Age (years)	227	61.1	10.7	63	54	68	24	85
Cumulative oxaliplatin dose (mg/m^2^)	211	611.1	188.2	658.8	500.0	765.0	63.8	1071.3
Number of consolidation chemotherapy cycles^e^	218	7.2	2.3	8	6	9	1	14
Duration of neoadjuvant treatment (months)	225	4.5	1.4	4.5	3.8	5.4	0.3	11.6
Follow‐up interval (months)	225	20.6	16.6	16	10	25	0	81
KPS prior to treatment (%)	227	91.3	9.1	90	90	100	60	100
KPS at follow‐up (%)	203	85.9	11.0	90	80	90	40	100

*Note*: Due to rounding, percentages may not total 100%. Missing data were treated using pairwise deletion, resulting in different numbers of included cases per variable.

Abbreviations: CRM, circumferential resection margin; CRT, chemoradiotherapy; EMVI, extramural vascular invasion; ESMO, European Society for Medical Oncology; LCRT, long‐course chemoradiotherapy; SCRT, short‐course radiotherapy.

^a^ Other fractionation schedules: 45.9 Gy (27 fractions) + simultaneous integrated boost to GTV: *n* = 1.

^b^ No concomitant chemotherapy in SCRT patients. Relative frequencies are thus referenced to *n* = 131 LCRT patients.

^c^ Other protocols of concomitant chemotherapy: Capecitabine p.o. (other dose than 1650 mg/m^2^/d): *n* = 3. Capecitabine + Oxaliplatin: *n* = 2.

^d^ Other protocols of sequential chemotherapy: 5‐FU monotherapy: *n* = 4. FOLFOXIRI: *n* = 1. Irinotecan/Capecitabine/Bevacizumab: *n* = 1.

^e^ Standardized to FOLFOX‐equivalents (q2w).

During treatment, 67 patients (29.5%) experienced severe toxicity (Table 2). Relevant CIPN during treatment was reported for 79 of 227 patients (35.6%) and, among them, severe CIPN for 13 patients (5.9%). The median interval to follow‐up was 16 (Q1–Q3: 10–25) months. At follow‐up, relevant CIPN was still reported for 61 of 227 patients (26.9%). Longitudinal comparison among 79 of 222 patients (35.6%) with relevant acute CIPN showed persistent relevant CIPN at follow‐up in 34 of them (43.0%; sect. S3 of Data S1). Furthermore, progressive CIPN, i.e., an increase from grade ≤1 during treatment to grade ≥2 at follow‐up, was observed in 26 of 143 patients (18.2%). The median cOXAd was higher in patients with relevant chronic CIPN (758.8 mg/m^2^ vs. 590.0 mg/m^2^, *p* < .001; sect. 4 of Data S1). Of note, the lowest reported dose associated with chronic CIPN was 130.0 mg/m^2^ only.

**TABLE 2:** Acute and chronic toxicity among included patients.

Acute toxicity			Chronic toxicity		
During treatment (CTCAE grades)			At follow‐up (CTCAE grades)		
CIPN (*N* = 222)	*n*	%	CIPN (*N* = 227)	*n*	%
None/Grade 0	73	32.9	None/Grade 0	98	43.2
Slight/Grade 1	70	31.5	Slight/Grade 1	68	30.0
Moderate/Grade 2	66	29.7	Moderate/Grade 2	53	23.3
Severe/Grade 3	13	5.9	Severe/Grade 3	8	3.5
Pain (*N* = 227)	*n*	%	Pain (*N* = 219)	*n*	%
None/Grade 0	94	43.5	None/Grade 0	158	72.1
Slight/Grade 1	68	31.5	Slight/Grade 1	40	18.3
Moderate/Grade 2	45	20.8	Moderate/Grade 2	16	7.3
Severe/Grade 3	9	4.2	Severe/Grade 3	5	2.3
Any type of toxicity (*N* = 227)	*n*	%	Any type of toxicity (*N* = 227)	*n*	%
None/Grade 0	2	0.9	None/Grade 0	38	16.7
Slight/Grade 1	36	16.0	Slight/Grade 1	84	37.0
Moderate/Grade 2	120	53.3	Moderate/Grade 2	70	30.8
Severe/Grade 3	53	23.6	Severe/Grade 3	35	15.4
Life‐threatening/Grade 4	14	6.2			

*Note*: Categorical variables are presented with absolute (*n*) and relative (%) frequencies of valid cases (*N*). Due to rounding, percentages may not total 100%. Missing data were treated using pairwise deletion, resulting in different numbers of included cases per variable.

Abbreviation: CIPN, chemotherapy‐induced peripheral neuropathy.

### 3.2 Dose‐effect relationship

**FIGURE 1:**
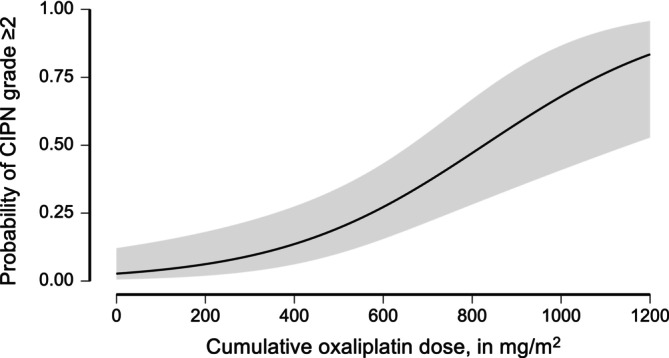
Predicted probability of relevant chronic chemotherapy‐induced polyneuropathy (CIPN) as a function of the cumulative oxaliplatin dose (cOXAd), based on a logistic regression model. See also sect. 2 and 6 of Data S1 for model specifications.

The cOXAd appeared to be a moderate classifier for symptomatic chronic CIPN in a ROC analysis (AUC = 0.700, 95% CI: 0.619–0.782, *p* < .001). Youden's Index J indicated an optimal cut‐off at a cOXAd of 654.38 mg/m^2^ (sect. 5 of Data S1). These results were confirmed by a multivariable logistic regression model, including 204 patients (Table 3). The multivariable logistic regression model, including prespecified clinical variables, significantly improved model fit over the null model with cOXAd only (Δ*χ* ^2^ = 13.27, Δdf = 5, *p* = .021). The model showed acceptable discrimination (AUC = 0.725) and calibration (Brier score = 0.175). No significant multicollinearity issues were observed (all variance influence factors [VIF] <1.2). Detailed model diagnostics and performance metrics are provided in sect. 6 of Data S1. Within this model, we found a statistically significant association of the cOXAd (adjusted OR 1.004, 95% CI: 1.002–1.007, *p* < .001) with the occurrence of symptomatic chronic CIPN (Figure 1). An additional FOLFOX cycle (+85 mg/m^2^ cOXAd) was associated with an adjusted OR of 1.446 (95% CI: 1.189–1.758). Based on the cohort baseline event rate (26.9%), the expected absolute risk increases from 26.9% to 34.7% (absolute increase 7.8%). The calculation of the expected risk increase for the cohort is provided in sect. 6 of Data S1. The likelihood of CIPN was about twofold increased in patients who required dose reduction during treatment (adjusted OR 2.008, 95% CI: 1.007–4.004, *p* = .048). Note that dose reduction had no significant influence on chronic CIPN in univariable regression modelling (*p* = .095). Contrarily, the follow‐up interval was significantly associated with a reduced likelihood of chronic CIPN in the univariable (*p* = .031), but not in the multivariable model (*p* = .250). Diabetes did not significantly influence the likelihood of CIPN (*p* = .651).

**TABLE 3:** Results from logistic regression analyses for the occurrence of relevant chronic chemotherapy‐induced polyneuropathy (CIPN) in relation to prespecified clinical variables.

Univariable logistic regression model (*n* = 203)					Multivariable logistic regression model (*n* = 203)			
Variable	OR	Lower 95% CI	Upper 95% CI	*p*‐value	OR	Lower 95% CI	Upper 95% CI	*p*‐value
Cumulative oxaliplatin dose (in mg/m^2^)	1.004	1.002	1.006	<.001	1.004	1.002	1.007	<.001
Age (in years)	0.981	0.953	1.010	.195	0.967	0.935	1.000	.051
Follow‐up interval (in months)	0.974	0.952	0.998	.031	0.982	0.955	1.010	.211
Sex (male)	0.710	0.362	1.392	.319	0.482	0.225	1.035	.061
Dose reduction required (yes)	1.696	0.912	3.154	.095	2.008	1.007	4.004	.048
Diabetes (present)	0.660	0.234	1.863	.433	0.769	0.246	2.403	.651

*Note*: Adjusted odds ratios (ORs) with 95% confidence intervals (CIs) and *p*‐values are presented from univariable and multivariable regression models. The number of patients (*n*) included after casewise deletion is provided.

### 3.3 HrQoL questionnaires

For this subgroup analysis, 67 QLQ‐C30 and 51 QLQ‐CIPN20 questionnaires were available. Exploratory non‐parametric group comparisons with the remaining sample (*n* = 176) revealed no statistically significant differences in sex distribution, presence of diabetes, prevalence of relevant CIPN at follow‐up, or cOXAd (all *p* > .05; sect. 7 of Data S1). However, patients who completed the HrQoL questionnaires more frequently experienced severe acute toxicity (*p* < .001), relevant chronic toxicity, and relevant acute CIPN (*p* = .041 and *p* = .027, respectively; neither remained significant after Holm–Bonferroni correction).

**FIGURE 2:**
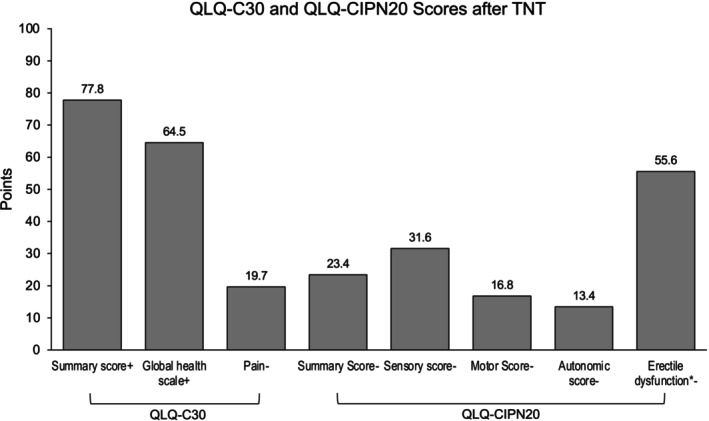
Mean scales of the QLQ‐C30 and QLQ‐CIPN20 questionnaires. Higher values indicate better function in functioning scales (+), whereas lower values indicate lower symptoms in the symptom scales (−). Detailed summary statistics are provided in sect. 8 of Data S1. Note that QLQ‐CIPN20 “Erectile Dysfunction” is only applicable to men (*).

A mean QLQ‐C30 GHS score of 64.5 (SD ±22.8) points and a QLQ‐CIPN20 summary score of 23.4 (SD ±18.5) points were reported (Figure 2) (sect. 8 of Data S1). The internal consistency of the QLQ‐CIPN20 scales was good (Cronbach's *α* ≥0.872 and McDonald's *ω* ≥0.875), except for the 2‐item Autonomic subscale, for which *ω* could not be estimated and *α* is reported (Cronbach's *α* = 0.583). Detailed test results are provided in sect. 9 of Data S1.

The QLQ‐CIPN20 Summary Score demonstrated good construct validity with all subscores (Spearman's *ρ* >0.77, *p* < .001; sect. 9 of Data S1). Convergent validity of the QLQ‐CIPN20 subscores was good, with moderate to strong negative correlations with the QLQ‐C30 Summary Score ranging from Spearman's *ρ* = −0.45 to −0.70 (all *p* ≤ .001), and moderate negative correlations with the QLQ‐C30 GHS ranging from Spearman's *ρ* = −0.39 to −0.57 (all *p* ≤ .005). The QLQ‐C30 “Pain” scores showed moderate to strong positive correlations with all QLQ‐CIPN20 subscores (Spearman's *ρ* = 0.41 to 0.61, all *p* ≤ .003). The QLQ‐CIPN20 “Erectile dysfunction” subscore correlated moderately with the QLQ‐C30 Summary Score (Spearman's *ρ* = −0.43, *p* = .013, not significant after Holm–Bonferroni correction). The corresponding test results are provided in sect. 10 of Data S1.

**FIGURE 3:**
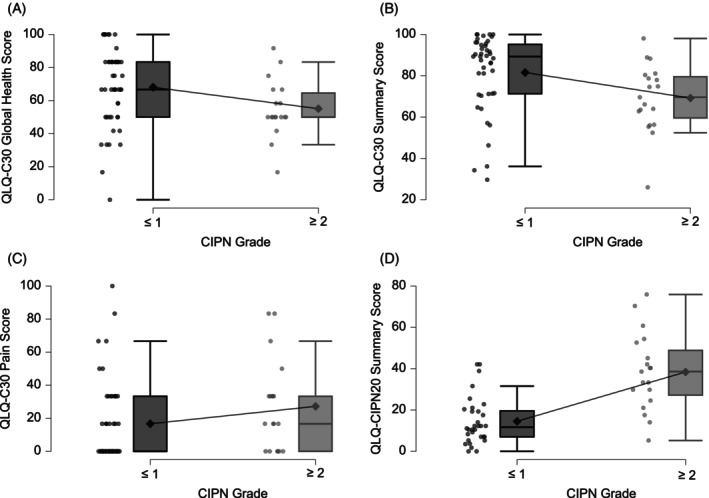
Distribution of QLQ‐C30 (A–C) and QLQ‐CIPN20 (D) scores stratified by presence of CIPN grade ≥2 at follow‐up. Boxplots illustrate medians and interquartile ranges; individual data points are overlaid to display score variability. Mean values are indicated by diamonds (♦) and connected between groups with a thin line to visualize mean differences. Note that higher values indicate better function in functioning scales (A, B), whereas lower values indicate lower symptoms in the symptom scales (C, D).

The QLQ‐CIPN Summary Score showed a high internal consistency (Cronbach's *α* = 0.933, McDonald's *ω* = 0.934) and correlated strongly with all component subscores (*ρ* ≥ 0.77, all *p* < .001), indicating that it reliably captures the information of the individual domains (sect. 9 and 10 of Data S1). Therefore, the Summary Score was used for subsequent correlation analyses and known‐group comparisons to reduce redundancy and limit multiple testing. As shown in sect. 11 of Data S1, there were only small differences (<10 points) in mean scores between men and women, as well as between patients with and without diabetes. Mean QLQ‐C30 Pain scores (−9.1 points, *d* = −0.36) and QLQ‐CIPN20 scores (−8.9 points, *d* = −0.49) were lower in men, not exceeding the predefined cut‐off (>10 points). Patients with clinically relevant CIPN at follow‐up reported lower QLQ‐C30 GHS (−13.0 points, Cohen's *d* = −0.59) and lower QLQ‐C30 Summary Scores (−12.4 points, *d* = −0.68). In contrast, these patients had higher QLQ‐C30 Pain Scores (+10.5 points, *d* = 0.42) and QLQ‐CIPN20 Summary Scores (+23.9 points, *d* = +1.65). A graphical comparison of these differences is provided in Figure 3.

## 4 DISCUSSION

To the best of our knowledge, this is the first analysis of oxaliplatin‐related CIPN within TNT of rectal cancer. While CIPN after oxaliplatin‐based chemotherapy has been widely investigated, its role and impact on HrQoL are not comprehensively studied. The herein evaluated QLQ‐CIPN20 module has not officially been validated for use in different entities and treatment settings, making this work relevant for the establishment of validated patient‐reported outcome measurements (PROMs). We found a high prevalence of relevant CIPN (grade ≥2) at follow‐up, and HrQoL was significantly affected in these patients. Of note, no distinct cut‐off for clinically relevant CIPN was identified, and symptoms occurred even after relatively low cumulative doses in some patients. The minimal cOXAd associated with relevant CIPN (130 mg/m^2^) was reached after only one CAPOX cycle. The presence of CIPN was associated with a large deterioration in the QLQ‐CIPN20 Summary Score (*d* = 1.58) and moderate impairments in QoL‐C30 domains (*d* = −0.56 and −0.68). Given the broad spectrum of CIPN‐related symptoms, its occurrence may impact several activities of daily living (ADLs), thereby affecting HrQoL overall.

The herein described long‐term TNT protocols lead to high CR‐rates of 48%.^27^ This might, at least in part, be associated with the high‐intense sequential chemotherapy protocols, as patients in our cohort received a median of 8 cycles of consolidation chemotherapy. We observed high rates (41%) of CIPN grade 1/2, which exceeds rates within the CAO/ARO/AIO‐12 trial (36%)^6^ and the CAO/ARO/AIO‐16 trial (29%).^28^ In these trials, 3 cycles of consolidation chemotherapy (FOLFOX) were administered after CRT with concomitant oxaliplatin, resulting in a lower scheduled cOXAd of 455 mg/m^2^. Contrarily, CIPN grade 1/2 was more frequent within the RAPIDO trial^4^ (79% after 9 cycles FOLFOX, i.e., 765 mg/m^2^) and the PRODIGE23 trial^5^ (83% after 6 cycles FOLFIRINOX plus 6 cycles FOLFOX, i.e., 1020 mg/m^2^). In both trials, higher cOXAd were scheduled compared with the CAO/ARO/AIO‐12/16 trials, exceeding reported thresholds. However, the prevalence of relevant chronic CIPN in our cohort aligns with findings from a systematic review reporting approximately 30% at 6 months follow‐up across different treatment schedules and neurotoxic agents.^15^

We found a statistically significant dose‐effect relationship between cOXAd and CIPN grade ≥2, which is consistent with earlier reports.^1^, ^29^ The estimated risk for relevant CIPN at follow‐up increased by 7.8% per additional FOLFOX cycle, representing a clinically meaningful elevation in risk. Longer follow‐up intervals were not associated with a reduced likelihood of CIPN. Recovery from CIPN is considered a long‐lasting process that may extend over several years.^1^, ^2^, ^3^ Moreover, the maximum severity of CIPN is often reported 2–3 months after discontinuation of oxaliplatin.^3^ Therefore, the follow‐up period in this cohort was likely too short to detect any meaningful associations. In contrast to the literature,^30^ the presence of diabetes was not associated with a significantly increased likelihood of CIPN in our cohort. We found a median cOXAd of 758.8 mg/m^2^ in patients with relevant CIPN at follow‐up, which aligns with the range of thresholds described in the literature (cOXAd 540–850 mg/m^2^ ^1^, ^2^, ^31^). The optimal cut‐off derived from ROC analysis (about 650 mg/m^2^) should, however, be interpreted with caution and requires external validation. Dose reduction during chemotherapy generally indicates the presence of dose‐limiting toxicity. Besides hematologic adverse events, CIPN is a common reason for treatment modification.^15^ Therefore, the observed association between dose reduction and higher CIPN prevalence likely represents reverse causality, as dose reductions are often implemented in response to emerging neuropathy rather than preventing it. This finding could also suggest that patients requiring dose adjustments may constitute a more vulnerable subgroup with a higher susceptibility to neurotoxicity.

Although overall HrQoL was not reduced in this cohort compared with a general colorectal cancer reference cohort, HrQoL was lower than in an age‐matched reference sample from the German general population.^32^ Given the relevant differences in mean HrQoL scores and the corresponding moderate to large effect sizes observed in our cohort, a detrimental impact of oxaliplatin‐related CIPN on HrQoL at follow‐up appears likely. Considering the substantial impairment and high symptom burden associated with CIPN, the established benefits of NOM for bowel function and HrQoL should be carefully weighed against the additional neurotoxicity of oxaliplatin. For example, the TIMING trial demonstrated increasing CR rates with a higher number of chemotherapy cycles.^33^ Contrarily, a pooled analysis of the CAO/ARO/AIO‐12 and OPRA trials did not show a survival benefit from the additional cycles provided in the OPRA trial.^11^ This aspect is of particular relevance in the light of the intended NOM, especially for non‐high‐risk patients. TNT may represent an overtreatment if no CR is achieved,^34^ and exceeds toxicity compared with standard CRT.^4^, ^35^

The QLQ‐CIPN20 module showed excellent internal consistency and construct validity in comparison with the QLQ‐C30. Moreover, the considerable correlations between the Summary Score and the Autonomic, Motor, and Sensory subscores support the use of this single multi‐item scale instead of the individual subscales, facilitating interpretability and reducing statistical issues related to multiple testing. In addition, we observed plausible correlations between clinical parameters and CIPN, further supporting the module's validity. Thus, we were able to replicate findings regarding the convergent validity observed with both oxaliplatin and other chemotherapy agents known to cause neuropathy.^21^, ^36^, ^37^, ^38^, ^39^

## 5 STRENGTHS AND LIMITATIONS

This multicenter study included 227 patients from 23 high‐volume centers across Germany and Austria, providing a broad real‐world cohort treated according to current TNT protocols. The inclusion criteria and statistical analyses were pre‐specified in the study protocol, ensuring methodological transparency. Moreover, the uniform treatment sequence, i.e., either LCRT or SCRT followed by consolidation chemotherapy, helped to reduce heterogeneity across centers. Reported potential confounders, defined a priori, were incorporated into the regression models to mitigate bias, thereby strengthening the validity of the observed associations.

Nevertheless, several limitations must be acknowledged. As a retrospective, non‐randomized analysis, causal inference is limited. Not all patients completed HrQoL questionnaires, which reduced statistical power for exploratory analyses. Respondents had slightly higher rates of CIPN and chronic toxicity. These differences were not significant after correction for multiple testing, but a potential selection bias toward patients with greater symptom burden cannot be excluded. Consequently, the generalizability to the overall patient population may be limited. The sample size further restricted subgroup analyses, particularly for the HrQoL evaluation. Consequently, prospective longitudinal studies with larger cohorts are needed to confirm these findings, assess test–retest reliability regarding the HrQoL questionnaires, and clarify the long‐term impact of oxaliplatin exposure on patient‐reported outcomes after TNT. Although no uniform standard for the use of LCRT or SCRT was applied in this sample, we would not expect differences in peripheral neuronal damage between the radiotherapy regimens, as these are considered local treatments. Furthermore, we have previously demonstrated that there was no statistically significant difference in overall toxicity between LCRT and SCRT.^27^

## 6 CONCLUSION

CIPN was a common chronic toxicity following TNT in this multicenter cohort, demonstrating a considerable association with cOXAd. Nearly half of the included patients with a cOXAd of approximately 750 mg developed neuropathy. The estimated risk increased by 7.8% per additional FOLFOX cycle in our cohort. The presence of chronic CIPN was associated with reduced general HrQoL and increased symptom scores for pain, as well as sensory, motor, and autonomic impairments. Given the increasing use of TNT in locally advanced rectal cancer, future prospective studies are warranted to determine the optimum number of consolidation chemotherapy cycles with respect to tumor control, toxicity, and HrQoL.

## AUTHOR CONTRIBUTIONS

**Georg W. Wurschi:** Conceptualization; investigation; data curation; methodology; formal analysis; project administration; writing – original draft; visualization. **Andreas Hinz:** Methodology; formal analysis; writing – original draft. **Melanie Schneider:** Investigation; writing – review and editing. **Jan‐Niklas Becker:** Investigation; writing – review and editing. **Bernd Frerker:** Investigation; writing – review and editing. **Samuel M. Vorbach:** Investigation; writing – review and editing. **Felix Ehret:** Investigation; writing – review and editing. **Markus Diefenhardt:** Investigation; writing – review and editing. **Fabian Schunn:** Investigation; writing – review and editing. **Maria‐Elena von Gruben:** Investigation; writing – review and editing. **Marcel Büttner:** Investigation; writing – review and editing. **Elgin Hoffmann:** Investigation; writing – review and editing. **Alexander Rühle:** Conceptualization; investigation; writing – review and editing. **Josephine Beier:** Investigation; writing – review and editing. **Simone Ferdinandus:** Investigation; writing – review and editing. **Maike Trommer:** Investigation; writing – review and editing. **Ezgi Ceren Sahin:** Investigation; writing – review and editing. **Julian Hlouschek:** Investigation; writing – review and editing. **Kynann Aninditha:** Investigation; writing – review and editing. **Daphne Schepers von Ohlen:** Investigation; writing – review and editing. **Justus Kaufmann:** Investigation; writing – review and editing. **Alina Depardon:** Investigation; writing – review and editing. **Hai Minh Ha:** Investigation; writing – review and editing. **Christopher Kessler:** Investigation; writing – review and editing. **Adrianna Cieslak:** Investigation; writing – review and editing. **Simon Trommer:** Investigation; writing – review and editing. **Alexander Fabian:** Investigation; writing – review and editing. **Florian Rißner:** Resources; project administration; writing – review and editing; software. **Maximilian Römer:** Investigation; writing – review and editing; validation. **Klaus Pietschmann:** Conceptualization; supervision; writing – review and editing.

## CONFLICT OF INTEREST STATEMENT

Felix Ehret has received honoraria and travel support from ZAP Surgical Systems, Inc. and Accuray, Inc., and acknowledges research funding from the German Cancer Aid and Accuray, Inc., all unrelated to the submitted work. Alexander Rühle received speaker fees, travel support and research grants from Novocure, consulting fees from Johnson&Johnson and Need Inc., speaker fees from Merck Healthcare Germany as well as from AstraZeneca, all outside the submitted work. Alexander Fabian received honoraria from Merck Sharp and Dohme. All other authors report having no conflict of interest regarding the submitted work.

## ETHICS STATEMENT

The study was approved by the local ethics committee of the Faculty of Medicine at Jena University Hospital (reference number: 2023‐3042‐Bef, amended to allow inclusion until 2024) and by each participating center's ethics committee. Written informed consent was not required for the primary retrospective analysis but was obtained from patients in the secondary cross‐sectional quality‐of‐life evaluation. The study adhered to the Declaration of Helsinki. The study protocol was prospectively registered with the German Clinical Trials Registry (DRKS, No. 00033000) and accredited by the radiation oncology working group of the German Cancer Society (Arbeitsgemeinschaft Radiologische Onkologie, ARO).

## Supporting information


**Data S1.** Supporting Information.

## Data Availability

The data that support the findings of this study are available on request from the corresponding author.
