# Supplementary material for: Oxaliplatin‐induced neuropathy after total neoadjuvant therapy for rectal cancer: Dose–response relationship and impact on quality of life
Source: Int J Cancer. 2026 Feb 18;159(1):198–209. doi: 10.1002/ijc.70396 (PMC13140008; doi:10.1002/ijc.70396)
Supplement: Supplementary file 1 — Data S1. Supporting Information. [file IJC-159-198-s001.pdf]

# **Supplementary Document**: Oxaliplatin-Induced Neuropathy after Total Neoadjuvant Therapy for Rectal Cancer: Dose-Response Relationship and Impact on Quality of Life

Georg W. Wurschi, Andreas Hinz, Melanie Schneider, Jan-Niklas Becker, Bernd Frerker, Samuel Vorbach, Felix Ehret, Markus Diefenhardt, Fabian Schunn, Maria-Elena von Gruben, Marcel Büttner, Elgin Hoffmann, Alexander Rühle, Josephine Beier, Simone Ferdinandus, Maike Trommer, Ezgi Ceren Sahin, Julian Hlouschek, Kynann Aninditha, Daphne Schepers von Ohlen, Justus Kaufmann, Alina Depardon, Hai Minh Ha, Simon Trommer, Christopher Kessler, Adrianna Cieslak, Alexander Fabian, Florian Rißner, Maximilian Römer and Klaus Pietschmann

## Table of contents

S1. Participating centers ........ 2

S2. Potential risk factors considered in the endpoint-specific regression modelling ........ 3

S3. Progression of Acute to Chronic CIPN ........ 4

S4. Comparison of oxaliplatin dose and follow-up intervals stratified by presence of CIPN ........ 5

S5. ROC Analysis: Oxaliplatin dose threshold ........ 6

S6. Regression modeling of chronic CIPN ........ 7

S7. Comparison of patients with versus without completed HrQoL questionnaires (sensitivity analysis) ........ 9

S8. Health-related quality of life scores of QLQ-C30 and QLQ-CIPN20 questionnaires ........ 14

S9. Internal consistency of QLQ-CIPN20 scores ........ 15

S10. Construct and convergent validity of the QLQ-CIPN20. ........ 17

S11. Known-group comparisons of QLQ-C30 and QLQ-CIPN20 scores ........ 18

# S1. Participating centers

| Center | Number of Patients |
|---|---|
| Jena University Hospital (Jena / Germany) | 37 |
| University Hospital Carl Gustav Carus Dresden (Dresden / Germany) | 27 |
| Hannover Medical School (Hannover / Germany) | 25 |
| Medical University of Innsbruck (Innsbruck / Austria) | 23 |
| Charité - Universitätsmedizin Berlin (Berlin / Germany) | 20 |
| Rostock University Medical Center (Rostock / Germany) | 19 |
| University Hospital Goethe University Frankfurt (Frankfurt (Main) / Germany) | 18 |
| University Hospital Hamburg-Eppendorf (Hamburg / Germany) | 11 |
| University Hospital Heidelberg (Heidelberg / Germany) | 9 |
| University Medical Center Schleswig-Holstein / Campus Lübeck (Lübeck / Germany) | 7 |
| University Medical Center of the Johannes-Gutenberg-University Mainz (Mainz / Germany) | 6 |
| University Hospital Tübingen (Tübingen / Germany) | 6 |
| Otto von Guericke Universität Magdeburg (Magdeburg / Germany) | 5 |
| Universitätsklinikum Erlangen, Friedrich-Alexander-Universität Erlangen-Nürnberg (Erlangen / Germany) | 3 |
| West German Cancer Center, University Hospital Essen (Essen / Germany) | 3 |
| Klinikum Stuttgart (Stuttgart / Germany) | 3 |
| University Medicine Mannheim, Medical Faculty Mannheim (Mannheim / Germany) | 2 |
| Technical University of Munich (TUM), School of Medicine and Klinikum Rechts der Isar (München / Germany) | 1 |
| University Medical Center Schleswig-Holstein / Campus Kiel (Kiel / Germany) | 1 |
| Faculty of Medicine and University Hospital Cologne (Köln / Germany) | 1 |
| Total | 227 |

## S2. Potential risk factors considered in the endpoint-specific regression modelling

For each endpoint, the considered potential risk factors are provided together with their unit and categories, respectively. Abbreviations: Chemotherapy-induced peripheral neuropathy, CIPN

| Primary endpoint | Potential risk factors |
|---|---|
| Relevant chronic CIPN (grade ≥2) | • Age, per additional year<br>• Male sex (versus female sex)<br>• Cumulative oxaliplatin dose, in $mg/m^2$<br>• Follow-up interval, in months<br>• Diabetes (present versus absent) |

# S3. Progression of Acute to Chronic CIPN

Crosstabulation of patients with relevant acute CIPN and subgroups stratified by the presence of relevant chronic CIPN. Absolute numbers (n) and relative frequencies (%) within each subgroup are reported. Percentages may not sum to 100% due to rounding.

| | | **Chronic CIPN** | | |
|---|---|---|---|---|
| | | Grade ≤ 1 | Grade ≥ 2 | Total |
| **Acute CIPN (grade ≤ 1)** | Count | 117 | 26 | 143 |
| % within acute CIPN grade ≥2 | | 81.8% | 18.2% | 100.0% |
| % within chronic CIPN grade ≥2 | | 72.2% | 43.3% | 64.4% |
| | | | | |
| **Acute CIPN (grade ≥ 2)** | Count | 45 | 34 | 79 |
| % within acute CIPN grade ≥2 | | 57.0% | 43.0% | 100.0% |
| % within chronic CIPN grade ≥2 | | 27.8% | 56.7% | 35.6% |
| | Count | 162 | 60 | 222 |
| % within acute CIPN grade ≥2 | | 73.0% | 27.0% | 100.0% |
| % within chronic CIPN grade ≥2 | | 100.0% | 100.0% | 100.0% |

# S4. Comparison of oxaliplatin dose and follow-up intervals stratified by presence of CIPN

Exploratory subgroup comparisons of cumulative oxaliplatin dose (cOXAd) and follow-up interval, stratified by chronic chemotherapy-induced peripheral neuropathy (CIPN) and corresponding non-parametric test statistics. Missing data were treated using pairwise deletion, resulting in different numbers of included cases per variable. Further abbreviations: Karnofsky Performance Status (KPS).

| | **Cumulative oxaliplatin dose, in mg/m$^2$** | | **Follow-up interval, in months** | |
|---|---|---|---|---|
| **CIPN at follow-up** | Grade ≤1 | Grade ≥2 | Grade ≤1 | Grade ≥2 |
| **Valid cases (n)** | 155 | 56 | 164 | 61 |
| **Mean** | 581.5 | 693.0 | 22.0 | 16.9 |
| **SD** | 183.8 | 177.0 | 18.5 | 9.0 |
| **Minimum** | 63.8 | 130.0 | 0.0 | 4.0 |
| **Maximum** | 1040.0 | 1071.3 | 81.0 | 38.0 |
| **Median** | 590.0 | 758.8 | 17.0 | 15.0 |
| **Q1** | 500.0 | 658.8 | 10.0 | 11.0 |
| **Q3** | 716.3 | 780.0 | 27.0 | 22.0 |
| **Mann-Whitney U test** | | | | |
| **U** | | 2602 | | 5411 |
| **p** | | <0.001* | | 0.346 |
| **Rank-Biserial Correlation** | | 0.400 | | -0.082 |
| **SE Rank-Biserial Correlation** | | 0.090 | | 0.087 |

Footnotes:
* Significant difference at unadjusted ($p < 0.05$) and Holm-Bonferroni-adjusted threshold.

# S5. ROC Analysis: Oxaliplatin dose threshold

Receiver Operating Characteristic (ROC) curve for cumulative oxaliplatin dose (cOXAd, in mg/m$^2$) predicting chronic CIPN grade ≥2. The area under the curve (AUC) with its 95% confidence interval is shown. The optimal threshold was determined using Youden's Index (J).

| **Area Under the Curve (AUC)** | | | | |
|---|---|---|---|---|
| **Area** | Std. Error | Asymptotic Significance Level | Asymptotic 95% Confidence Interval | |
| | | | Lower Bound | Upper Bound |
| **0.700** | 0.042 | <0.001 | 0.619 | 0.782 |

| **cOXAd, in mg/m$^2$** | **Sensitivity** | **1 – Specificity** | **Youden's Index J** |
|---|---|---|---|
| **654.38** | 0.768 | 0.419 | 1.349 |

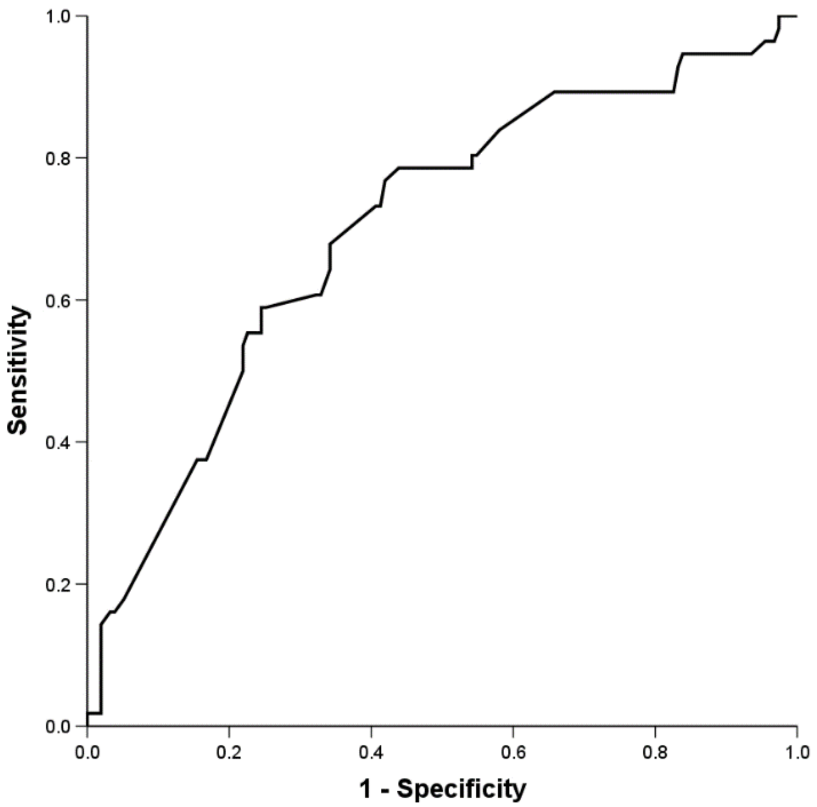


Diagonal segments are produced by ties.

# S6. Regression modeling of chronic CIPN

Additional performance metrics and model diagnostics for the multivariable logistic regression model predicting symptomatic chronic chemotherapy-induced polyneuropathy (CIPN). Included are classification metrics (accuracy, sensitivity, specificity, precision, F-measure, H-measure), pseudo-$R^2$ values (McFadden, Nagelkerke, Tjur, Cox & Snell), deviance, information criteria (AIC, BIC), and multicollinearity diagnostics (tolerance, variance inflation factor). Furthermore, the calculation of expected risk increase for the baseline cohort is provided.

| Model | Deviance | AIC | BIC | df | $\Delta X^2$ | p | McFadden $R^2$ | Nagelkerke $R^2$ | Tjur $R^2$ | Cox & Snell $R^2$ |
|---|---|---|---|---|---|---|---|---|---|---|
| $M_0$ | 224.3 | 228.3 | 234.9 | 201 | | | 0.00 | 0.00 | 0.08 | 0.00 |
| $M_1$ | 211.0 | 225.0 | 248.2 | 196 | 13.268 | 0.021 | 0.059 | 0.095 | 0.127 | 0.063 |

Footnotes:
$M_0$ includes cumulative oxaliplatin dose
$M_1$ includes cumulative oxaliplatin dose, age, follow-up time point, sex, dose reduction, diabetes

| Multicollinearity Diagnostics | | |
|---|---|---|
| | Tolerance | VIF |
| **Cumulative oxaliplatin dose (cOXAd)** | 0.862 | 1.159 |
| **Age** | 0.877 | 1.140 |
| **Follow-up timepoint** | 0.981 | 1.019 |
| **Sex** | 0.903 | 1.107 |
| **Dose reduction** | 0.914 | 1.094 |
| **Diabetes** | 0.959 | 1.043 |

| Performance metrics | |
|---|---|
| | Value |
| **Accuracy** | 0.739 |
| **AUC** | 0.725 |
| **Sensitivity** | 0.179 |
| **Specificity** | 0.952 |
| **Precision** | 0.588 |
| **F-measure** | 0.274 |
| **Brier score** | 0.175 |
| **H-measure** | 0.217 |

**Calculation of the expected risk increase ΔP for an additional FOLFOX cycle in the baseline cohort.**

Given the baseline probability of CIPN grade ≥2 ($P_0$ = 0.269) and the Odds Ratio (OR) of 1.446 for an 85 mg/m$^2$ increase in cumulative oxaliplatin dose (cOXAd), ΔP was calculated using the following formulas.

$$Odds_0 = \frac{P_0}{1-P_0} \; and \; OR = \frac{Odds_1}{Odds_0} \qquad (1)$$

$$P_1 = \frac{Odds_1}{1 + Odds_1} = \frac{OR \times Odds_0}{1 + OR \times Odds_0} = \frac{OR \times \frac{P_0}{1 - P_0}}{1 + OR \times \frac{P_0}{1 - P_0}} = 0.347 \quad (2)$$

$$\Delta p = p_1 - p_0 = 0.347 - 0.269 \quad (3)$$

$$\Delta p = \frac{OR \times \frac{P_0}{1 - P_0}}{1 + OR \times \frac{P_0}{1 - P_0}} - P_0 = 0.078 \quad (4)$$

## S7. Comparison of patients with versus without completed HrQoL questionnaires (sensitivity analysis)

Comparison demographics, clinical characteristics, and outcomes, stratified by the completion of QLQ-C30 and QLQ-CIPN20 questionnaires. Chi-squared and Mann-Whitney U tests were used. Holm-Bonferroni-adjusted significance was determined using step-down correction for multiple comparisons. Bar charts of relative frequencies (%) are provided for visualization.

| **Contingency Tables** | | | | | | | |
|---|---|---|---|---|---|---|---|
| | HrQoL questionnaires available? | | | | | | |
| **Sex** | Not available | Available | Total | Chi-Squared Tests | | | |
| **Female (f)** | 50 | 13 | 63 | | Value | df | p |
| **Male (m)** | 126 | 38 | 164 | $X^2$ | 0.168 | 1 | 0.682 |
| **Total** | 176 | 51 | 227 | N | 227 | | |
| **Diabetes** | Not available | Available | Total | | | | |
| **No** | 154 | 45 | 199 | | Value | df | p |
| **Yes** | 21 | 6 | 27 | $X^2$ | 0.002 | 1 | 0.964 |
| **Total** | 175 | 51 | 226 | N | 226 | | |
| **Acute toxicity (any type)** | Not available | Available | Total | | Value | df | p |
| **Grade ≤ 1** | 134 | 24 | 158 | $X^2$ | 16.922 | 1 | < 0.001** |
| **Grade ≥ 2** | 40 | 27 | 67 | N | 225 | | |
| **Total** | 174 | 51 | 225 | | | | |
| **Acute CIPN** | Not available | Available | Total | | Value | df | p |
| **Grade ≤ 1** | 118 | 25 | 143 | $X^2$ | 4.921 | 1 | 0.027* |
| **Grade ≥ 2** | 55 | 24 | 79 | N | 222 | | |
| **Total** | 173 | 49 | 222 | | | | |
| **Chronic toxicity (any type)** | Not available | Available | Total | | Value | df | p |
| **Grade ≤ 1** | 101 | 21 | 122 | $X^2$ | 4.179 | 1 | 0.041* |
| **Grade ≥ 2** | 75 | 30 | 105 | N | 227 | | |
| **Total** | 176 | 51 | 227 | | | | |
| **Chronic CIPN** | Not available | Available | Total | | Value | df | p |
| **Grade ≤ 1** | 134 | 32 | 166 | $X^2$ | 3.608 | 1 | 0.057 |
| **Grade ≥ 2** | 42 | 19 | 61 | N | 227 | | |
| **Total** | 176 | 51 | 227 | | | | |

Footnotes:

* significant difference at unadjusted threshold (p<0.05).

** Significant difference at unadjusted (p < 0.05) and Holm-Bonferroni-adjusted threshold.

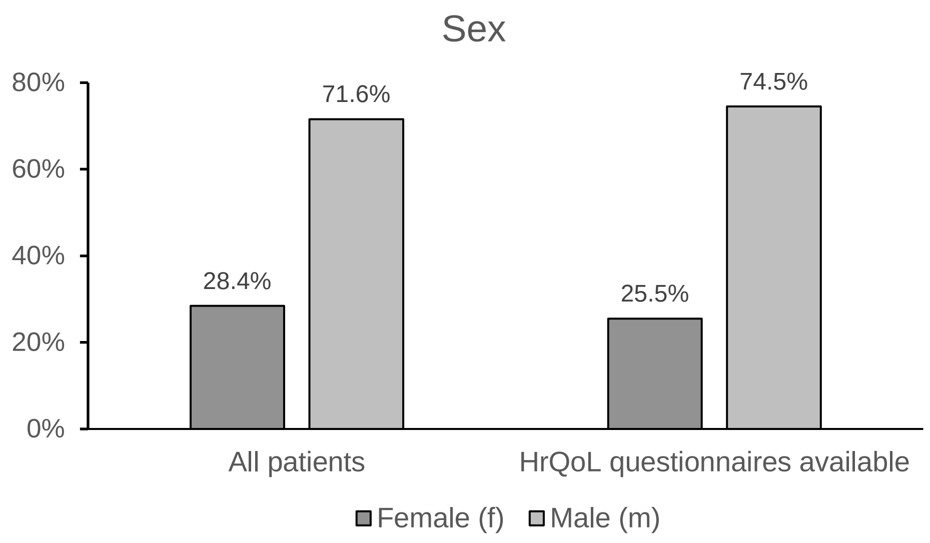
Sex
80%
60%
40%
20%
0%
71.6%
28.4%
74.5%
25.5%
All patients
HrQoL questionnaires available
Female (f)
Male (m)

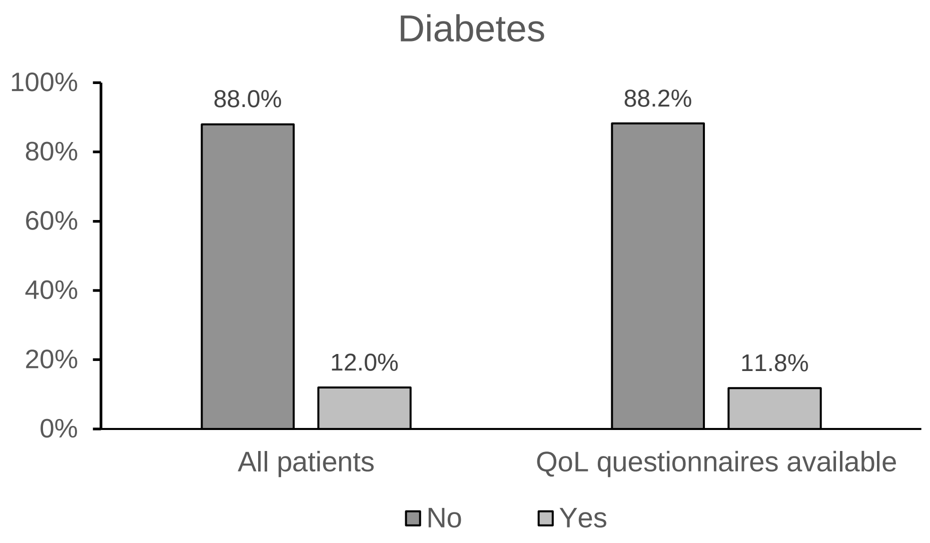
Diabetes
100%
80%
60%
40%
20%
0%
88.0%
12.0%
88.2%
11.8%
All patients
QoL questionnaires available
No
Yes

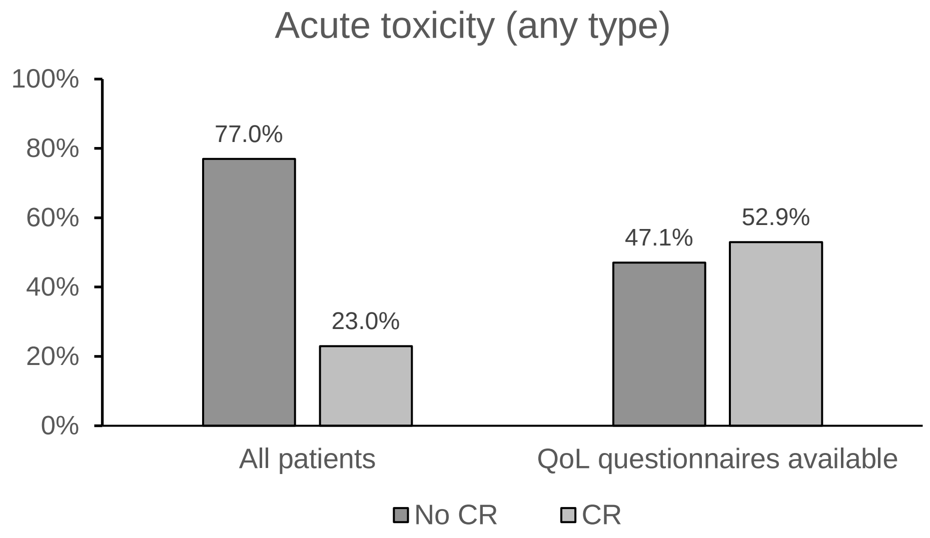
Acute toxicity (any type)
100%
80%
60%
40%
20%
0%
77.0%
23.0%
47.1%
52.9%
All patients
QoL questionnaires available
No CR
CR

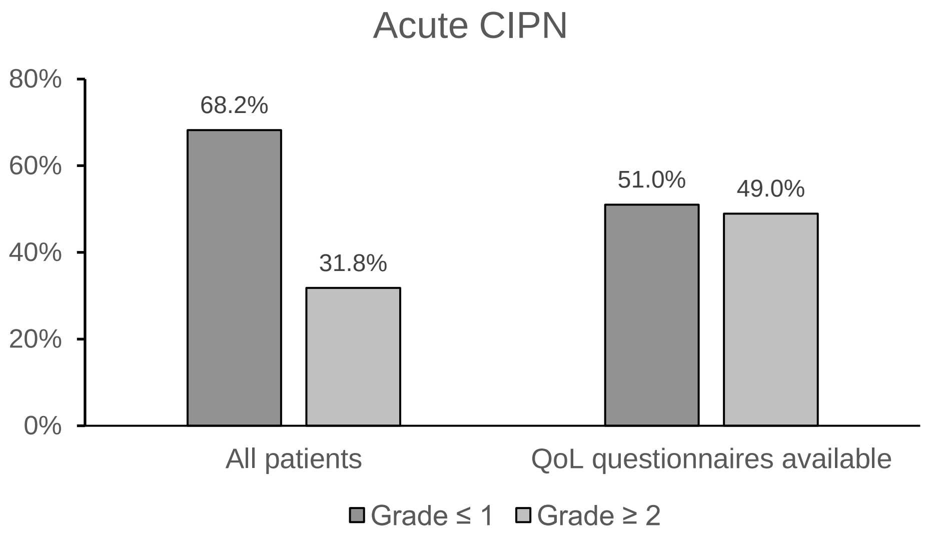
Acute CIPN
80%
60%
40%
20%
0%
68.2%
31.8%
51.0%
49.0%
All patients
QoL questionnaires available
Grade ≤ 1
Grade ≥ 2

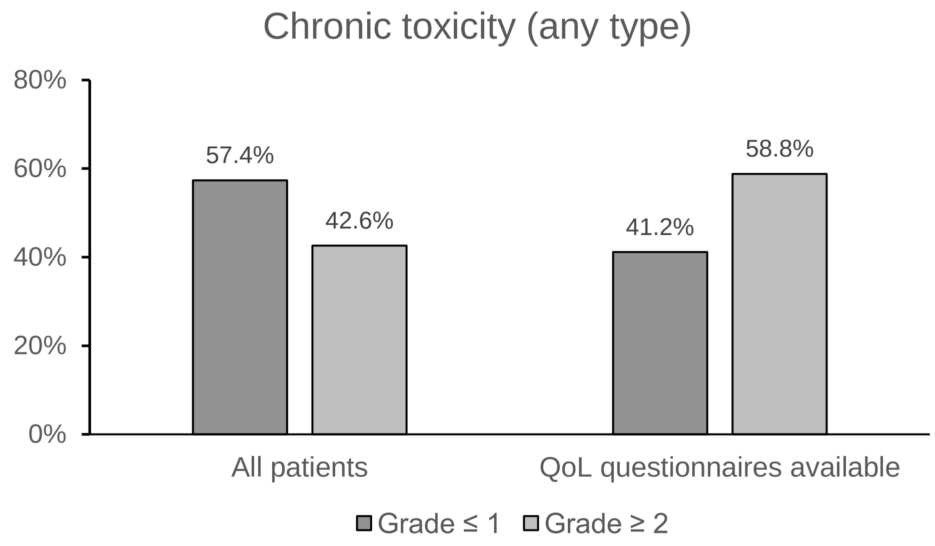
Chronic toxicity (any type)
80%
60%
40%
20%
0%
57.4%
42.6%
41.2%
58.8%
All patients
QoL questionnaires available
Grade ≤ 1
Grade ≥ 2

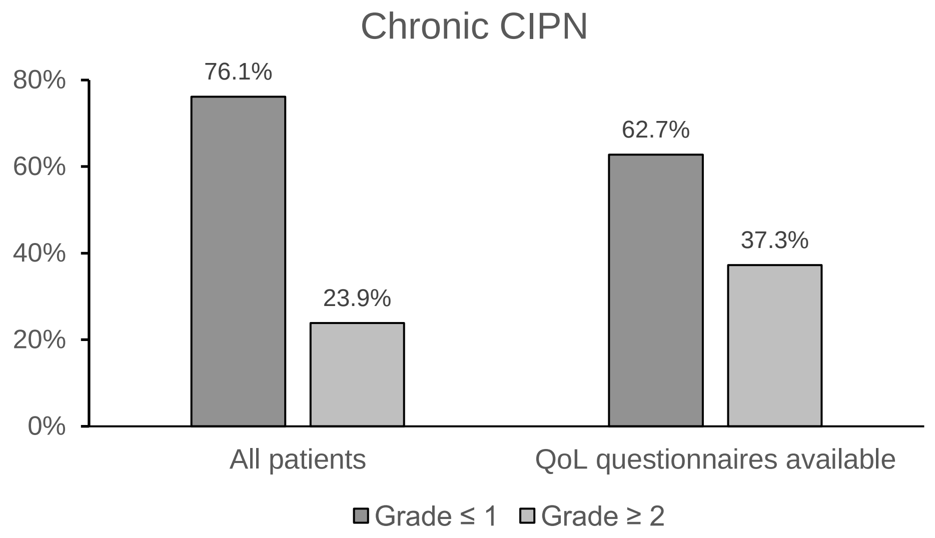
Chronic CIPN
80%
60%
40%
20%
0%
76.1%
23.9%
62.7%
37.3%
All patients
QoL questionnaires available
Grade ≤ 1
Grade ≥ 2

Continuous variables, stratified by the completion of QLQ-C30 and QLQ-CIPN20 questionnaires.

| | **Age, in years** | | **Cumulative oxaliplatin dose, in mg/m²** | | **KPS at follow-up, in %** | |
|---|---|---|---|---|---|---|
| **HRQoL questionnaires** | not available | available | not available | available | not available | available |
| **n** | 176 | 51 | 162 | 49 | 154 | 49 |
| **Mean** | 60.1 | 64.4 | 602.7 | 639.1 | 86.1 | 85.3 |
| **SD** | 10.8 | 9.5 | 191.9 | 174.4 | 11.5 | 9.4 |
| **Median** | 61 | 65 | 650.0 | 688.8 | 90 | 80 |
| **Q1** | 53 | 59 | 500.0 | 510.0 | 80 | 80 |
| **Q3** | 67 | 71 | 765.0 | 773.8 | 90 | 90 |
| **Minimum** | 24 | 38 | 63.8 | 170.0 | 40 | 70 |
| **Maximum** | 85 | 82 | 1071.3 | 901.3 | 100 | 100 |

Mann-Whitney-U test for continuous variables, stratified by the completion of QLQ-C30 and QLQ-CIPN20 questionnaires.

| | **U** | **p** | **Rank-Biserial Correlation** | **SE Rank-Biserial Correlation** |
|---|---|---|---|---|
| **Age, in years** | 3399 | 0.008* | 0.243 | 0.092 |
| **Cumulative oxaliplatin dose, in mg/m²** | 3506 | 0.216 | 0.117 | 0.094 |
| **KPS at follow-up, in %** | 4130 | 0.299 | -0.095 | 0.095 |

Footnotes:
* Significant difference at unadjusted ($p < 0.05$) and Holm-Bonferroni-adjusted threshold.

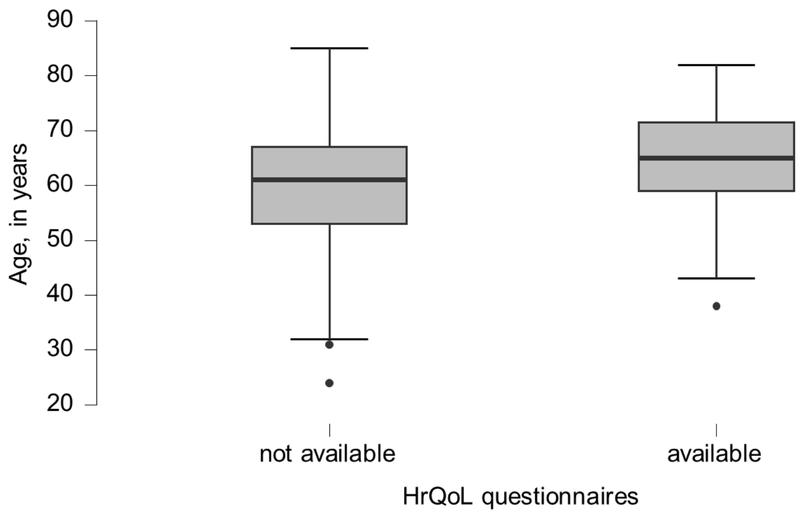
90
80
70
60
50
40
30
20
Age, in years
not available
available
HrQoL questionnaires

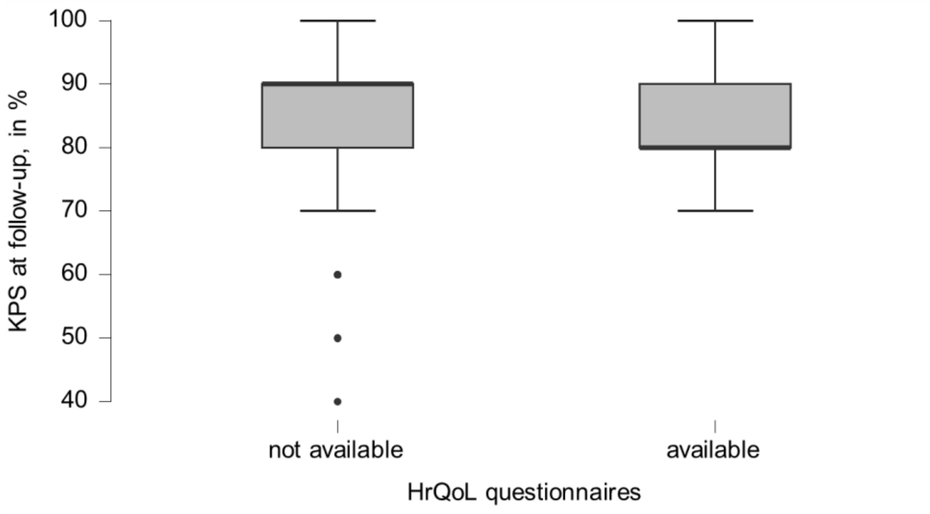
100
90
80
70
60
50
40
KPS at follow-up, in %
not available
available
HrQoL questionnaires

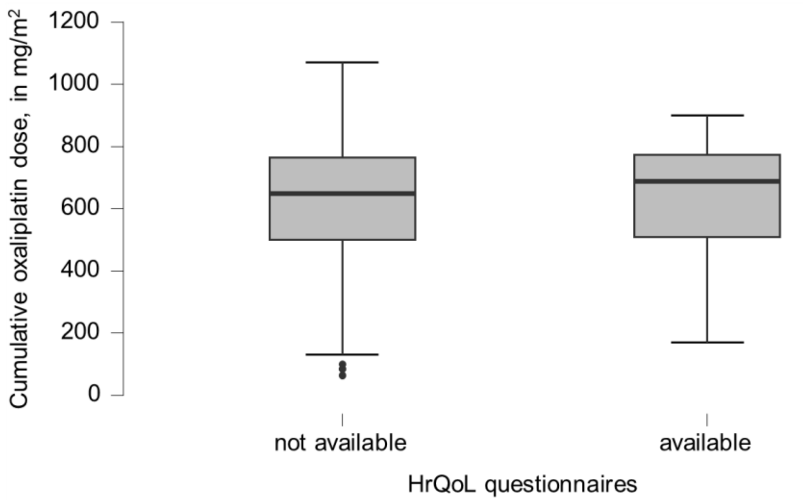
1200
1000
800
600
400
200
0
Cumulative oxaliplatin dose, in mg/m2
not available
available
HrQoL questionnaires

# S8. Health-related quality of life scores of QLQ-C30 and QLQ-CIPN20 questionnaires

Absolute frequencies (n), mean together with standard deviation (SD), and median together with 1st (Q1) and 3rd (Q3) quartile are provided. Higher values indicate better function in functioning scores[1], whereas lower values indicate lower symptoms in the symptom scores[2]. Missing data were treated using pairwise deletion, resulting in different numbers of included cases per variable.

| | QLQ-C30 | | | QLQ-CIPN20 | | | | |
|---|---|---|---|---|---|---|---|---|
| | Summary score[1] | Global health scale[1] | Pain[2] | Summary Score[2] | Sensory score[2] | Motor Score[2] | Autonomic score[2] | Erectile dysfunction[2,3] |
| **n** | 62 | 66 | 67 | 51 | 51 | 51 | 51 | 33 |
| **Mean** | 77.8 | 64.5 | 19.7 | 23.4 | 31.6 | 16.8 | 13.4 | 55.6 |
| **SD** | 18.9 | 22.8 | 25.6 | 18.5 | 21.9 | 18.2 | 18.9 | 40.5 |
| **Median** | 81.8 | 66.7 | 16.7 | 19.3 | 29.6 | 12.5 | 0.0 | 66.7 |
| **Q1** | 65.1 | 50.0 | 0.0 | 9.9 | 14.8 | 4.2 | 0.0 | 33.3 |
| **Q3** | 92.1 | 83.3 | 33.3 | 36.0 | 42.6 | 26.8 | 33.3 | 100.0 |

Footnotes:
1 Functioning score
2 Symptom score
3 Item only applicable to men.

## S9. Internal consistency of QLQ-CIPN20 scores

Assessment of internal consistency of the „Summary Scale“ (A, items 31-49), „sensory score“ (B, items 31-36, 39, 40, 48), „Motor Scale“ (C, items 37+38, 41-45, 49) with Cronbach’s α and McDonald’s ω. As the “Autonomic Scale“ (D) only comprised items 46+47, internal consistency was evaluated with Cronbach’s α only. Cronbach’s α and McDonald’s ω were assessed in the context of the influence of dropping single items from the scale (B). 95% confidence intervals were obtained from 1000 bootstrap samples.

(A)

| **Unidimensional Reliability: QLQ-CIPN20 Summary Score** | | | | |
|---|---|---|---|---|
| | | | 95% CI | |
| **Coefficient** | Estimate | Std. Error | Lower | Upper |
| **Coefficient ω** | 0.934 | 0.014 | 0.901 | 0.948 |
| **Coefficient α** | 0.933 | 0.013 | 0.902 | 0.951 |
| **Average interitem correlation** | 0.422 | 0.047 | 0.327 | 0.507 |

| **Individual Item Reliability Statistics** | | | | | | |
|---|---|---|---|---|---|---|
| | Coefficient ω (if item dropped) | | | Coefficient α (if item dropped) | | |
| **Item** | Estimate | Lower 95% CI | Upper 95% CI | Estimate | Lower 95% CI | Upper 95% CI |
| **CIPN Item 31** | 0.931 | 0.897 | 0.948 | 0.929 | 0.898 | 0.949 |
| **CIPN Item 32** | 0.931 | 0.900 | 0.946 | 0.929 | 0.896 | 0.949 |
| **CIPN Item 33** | 0.931 | 0.898 | 0.948 | 0.929 | 0.896 | 0.948 |
| **CIPN Item 34** | 0.931 | 0.897 | 0.946 | 0.929 | 0.895 | 0.949 |
| **CIPN Item 35** | 0.928 | 0.892 | 0.946 | 0.926 | 0.893 | 0.946 |
| **CIPN Item 36** | 0.927 | 0.892 | 0.945 | 0.925 | 0.892 | 0.946 |
| **CIPN Item 37** | 0.931 | 0.899 | 0.946 | 0.929 | 0.898 | 0.947 |
| **CIPN Item 38** | 0.931 | 0.901 | 0.947 | 0.929 | 0.899 | 0.948 |
| **CIPN Item 39** | 0.930 | 0.891 | 0.945 | 0.928 | 0.894 | 0.947 |
| **CIPN Item 40** | 0.932 | 0.896 | 0.949 | 0.930 | 0.896 | 0.950 |
| **CIPN Item 41** | 0.929 | 0.893 | 0.946 | 0.927 | 0.894 | 0.947 |
| **CIPN Item 42** | 0.929 | 0.893 | 0.945 | 0.927 | 0.895 | 0.947 |
| **CIPN Item 43** | 0.929 | 0.893 | 0.945 | 0.927 | 0.894 | 0.947 |
| **CIPN Item 44** | 0.931 | 0.901 | 0.945 | 0.929 | 0.899 | 0.947 |
| **CIPN Item 45** | 0.931 | 0.902 | 0.945 | 0.930 | 0.898 | 0.949 |
| **CIPN Item 46** | 0.932 | 0.898 | 0.948 | 0.930 | 0.897 | 0.950 |
| **CIPN Item 47** | 0.932 | 0.895 | 0.950 | 0.931 | 0.896 | 0.951 |
| **CIPN Item 48** | 0.937 | 0.903 | 0.951 | 0.936 | 0.900 | 0.955 |
| **CIPN Item 49** | 0.933 | 0.900 | 0.954 | 0.932 | 0.896 | 0.952 |

(B)

| **Unidimensional Reliability: QLQ-CIPN20 Sensory Score** | | | | |
|---|---|---|---|---|
| | | | 95% CI | |
| **Coefficient** | Estimate | Std. Error | Lower | Upper |
| **Coefficient ω** | 0.878 | 0.027 | 0.815 | 0.915 |
| **Coefficient α** | 0.874 | 0.029 | 0.804 | 0.910 |

| Individual Item Reliability Statistics | | | | | | |
|---|---|---|---|---|---|---|
| | Coefficient ω (if item dropped) | | | Coefficient α (if item dropped) | | |
| **Item** | Estimate | Lower 95% CI | Upper 95% CI | Estimate | Lower 95% CI | Upper 95% CI |
| **CIPN Item 31** | 0.865 | 0.799 | 0.905 | 0.858 | 0.784 | 0.899 |
| **CIPN Item 32** | 0.865 | 0.789 | 0.908 | 0.858 | 0.772 | 0.900 |
| **CIPN Item 33** | 0.864 | 0.800 | 0.905 | 0.858 | 0.783 | 0.899 |
| **CIPN Item 34** | 0.865 | 0.793 | 0.906 | 0.858 | 0.779 | 0.900 |
| **CIPN Item 35** | 0.857 | 0.783 | 0.902 | 0.852 | 0.769 | 0.897 |
| **CIPN Item 36** | 0.853 | 0.773 | 0.898 | 0.847 | 0.759 | 0.893 |
| **CIPN Item 39** | 0.858 | 0.789 | 0.901 | 0.852 | 0.771 | 0.894 |
| **CIPN Item 40** | 0.870 | 0.804 | 0.911 | 0.865 | 0.792 | 0.905 |
| **CIPN Item 48** | 0.889 | 0.826 | 0.929 | 0.888 | 0.822 | 0.928 |

(C)

| Unidimensional Reliability: QLQ-CIPN20 Motor Score | | | | |
|---|---|---|---|---|
| | | | 95% CI | |
| **Coefficient** | Estimate | Std. Error | Lower | Upper |
| **Coefficient ω** | 0.875 | 0.035 | 0.790 | 0.923 |
| **Coefficient α** | 0.872 | 0.035 | 0.784 | 0.917 |

| Individual Item Reliability Statistics | | | | | | |
|---|---|---|---|---|---|---|
| | Coefficient ω (if item dropped) | | | Coefficient α (if item dropped) | | |
| **Item** | Estimate | Lower 95% CI | Upper 95% CI | Estimate | Lower 95% CI | Upper 95% CI |
| **CIPN Item 37** | 0.858 | 0.768 | 0.910 | 0.855 | 0.759 | 0.904 |
| **CIPN Item 38** | 0.860 | 0.781 | 0.915 | 0.856 | 0.769 | 0.906 |
| **CIPN Item 41** | 0.848 | 0.749 | 0.906 | 0.845 | 0.739 | 0.898 |
| **CIPN Item 42** | 0.856 | 0.757 | 0.911 | 0.854 | 0.751 | 0.904 |
| **CIPN Item 43** | 0.853 | 0.757 | 0.907 | 0.851 | 0.746 | 0.901 |
| **CIPN Item 44** | 0.866 | 0.776 | 0.915 | 0.863 | 0.770 | 0.908 |
| **CIPN Item 45** | 0.857 | 0.767 | 0.910 | 0.854 | 0.757 | 0.902 |
| **CIPN Item 49** | 0.876 | 0.763 | 0.935 | 0.875 | 0.757 | 0.935 |

(D)

| Unidimensional Reliability: QLQ-CIPN20 Autonomic Score (Item 46 + 47) | | | | |
|---|---|---|---|---|
| | | | 95% CI | |
| **Coefficient** | Estimate | Std. Error | Lower | Upper |
| **Coefficient ω[1]** | - | - | - | - |
| **Coefficient α[1]** | 0.583 | 0.244 | -0.043 | 0.863 |
| **Pearson's r[1]** | 0.416 | | 0.155 | 0.622 |

Footnotes:
1 ω not reported: With only two items, the factor model is under identified and ω ≈ α. Pearson's correlation (r) is provided as a substitute measure of internal consistency.

# S10. Construct and convergent validity of the QLQ-CIPN20.

Spearman’s correlations assessing construct validity of QLQ-CIPN20 subscores (A) and convergent validity with QLQ-C30 scores (B). Confidence intervals are based on 1000 bootstrap replicates. Holm-Bonferroni-adjusted significance was determined using step-down correction for multiple comparisons. Higher values indicate better function in functioning scores, whereas lower values indicate lower symptoms in the symptom scores.

(A)

**Spearman's Correlations**

| | | | Spearman's ρ | p | Lower 95% CI | Upper 95% CI |
|---|---|---|---|---|---|---|
| **QLQ-CIPN20 Summary Score** | - | QLQ-CIPN20 Sensory Score | 0.960 | < 0.001* | 0.919 | 0.979 |
| | - | QLQ-CIPN20 Motor Score | 0.870 | < 0.001* | 0.741 | 0.935 |
| | - | QLQ-CIPN20 Autonomic Score | 0.771 | < 0.001* | 0.657 | 0.846 |

Footnotes:
* Significant difference at unadjusted (p < 0.05) and Holm-Bonferroni-adjusted threshold.

(B)

**Spearman's Correlations**

| | | | Spearman's ρ | p | Lower 95% CI | Upper 95% CI |
|---|---|---|---|---|---|---|
| **QLQ-C30 Summary Score** | - | QLQ-CIPN20 Summary Score | -0.698 | < 0.001** | -0.822 | -0.490 |
| **QLQ-C30 Summary Score** | - | QLQ-CIPN20 Sensory Score | -0.658 | < 0.001** | -0.797 | -0.461 |
| **QLQ-C30 Summary Score** | - | QLQ-CIPN20 Motor Score | -0.700 | < 0.001** | -0.836 | -0.497 |
| **QLQ-C30 Summary Score** | - | QLQ-CIPN20 Autonomic Score | -0.446 | 0.001** | -0.659 | -0.162 |
| **QLQ-C30 Global Health Scale** | - | QLQ-CIPN20 Summary Score | -0.484 | < 0.001** | -0.706 | -0.197 |
| **QLQ-C30 Global Health Scale** | - | QLQ-CIPN20 Sensory Score | -0.407 | 0.003* | -0.646 | -0.129 |
| **QLQ-C30 Global Health Scale** | - | QLQ-CIPN20 Motor Score | -0.568 | < 0.001** | -0.771 | -0.302 |
| **QLQ-C30 Global Health Scale** | - | QLQ-CIPN20 Autonomic Score | -0.393 | 0.005* | -0.596 | -0.127 |
| **QLQ-C30 Pain** | - | QLQ-CIPN20 Summary Score | 0.607 | < 0.001** | 0.369 | 0.791 |
| **QLQ-C30 Pain** | - | QLQ-CIPN20 Sensory Score | 0.558 | < 0.001** | 0.304 | 0.749 |
| **QLQ-C30 Pain** | - | QLQ-CIPN20 Motor Score | 0.550 | < 0.001** | 0.307 | 0.750 |
| **QLQ-C30 Pain** | - | QLQ-CIPN20 Autonomic Score | 0.411 | 0.003** | 0.153 | 0.653 |
| **QLQ-C30 Summary Score** | - | QLQ-CIPN20 Erectile dysfunction*** | -0.434 | 0.013* | -0.709 | -0.094 |
| **QLQ-C30 Global Health Scale** | - | QLQ-CIPN20 Erectile dysfunction*** | -0.347 | 0.052 | -0.620 | -0.018 |
| **QLQ-C30 Pain** | - | QLQ-CIPN20 Erectile dysfunction*** | 0.181 | 0.313 | -0.180 | 0.500 |

Footnotes:
* Significant difference at unadjusted threshold (p<0.05).
** Significant difference at unadjusted (p < 0.05) and Holm-Bonferroni-adjusted threshold.
*** Item only applicable to men.

## S11. Known-group comparisons of QLQ-C30 and QLQ-CIPN20 scores

Health-related quality of life scores of QLQ-C30 and QLQ-CIPN20 questionnaires stratified by clinical parameters. For each subgroup, valid cases (n), mean ± standard deviation (SD) are reported. Differences in means are presented together with the corresponding effect sizes (Cohen's d). Higher values indicate better function in functioning scores, whereas lower values indicate lower symptoms in the symptom scores.

| | **CIPN at follow-up** | | | | | | | |
|---|---|---|---|---|---|---|---|---|
| | QLQ-C30 Global Health Scale | | QLQ-C30 Summary Score | | QLQ-C30 Pain Scale | | QLQ-CIPN20 Summary Score | |
| **CTCAE grade** | ≤ 1 | ≥ 2 | ≤ 1 | ≥ 2 | ≤ 1 | ≥ 2 | ≤ 1 | ≥ 2 |
| **n** | 48 | 18 | 43 | 19 | 48 | 19 | 32 | 19 |
| **Mean** | 68.1 | 55.1 | 81.6 | 69.2 | 16.7 | 27.2 | 14.5 | 38.4 |
| **SD** | 23.7 | 17.4 | 18.9 | 16.4 | 24.6 | 27.3 | 11.4 | 18.7 |
| **Δ Mean** | | -13.0 | | -12.4 | | 10.5 | | 23.9 |
| **Cohen's d** | | -0.59 | | -0.68 | | 0.42 | | 1.65 |

| | **Sex (female versus male)** | | | | | | | | **Diabetes (absent versus present)** | | | | | | | |
|---|---|---|---|---|---|---|---|---|---|---|---|---|---|---|---|---|
| | QLQ-C30 Global Health Scale | | QLQ-C30 Summary Score | | QLQ-C30 Pain Scale | | QLQ-CIPN20 Summary Score | | QLQ-C30 Global Health Scale | | QLQ-C30 Summary Score | | QLQ-C30 Pain Scale | | QLQ-CIPN20 Summary Score | |
| | Female | Male | Female | Male | Female | Male | Female | Male | Absent | Present | Absent | Present | Absent | Present | Absent | Present |
| **n** | 17 | 49 | 16 | 46 | 17 | 50 | 13 | 38 | 59 | 7 | 56 | 6 | 45 | 6 | 60 | 7 |
| **Mean** | 59.8 | 66.2 | 74.5 | 78.9 | 26.5 | 17.3 | 30.0 | 21.2 | 65.3 | 58.3 | 77.8 | 77.4 | 20.6 | 11.9 | 23.0 | 22.4 |
| **SD** | 24.2 | 22.3 | 20.0 | 18.6 | 29.5 | 24.0 | 22.3 | 16.8 | 22.1 | 29.3 | 19.5 | 13.5 | 26.6 | 12.6 | 19.5 | 10.7 |
| **Δ Mean** | | 6.4 | | 4.4 | | -9.1 | | -8.9 | | -6.9 | | -0.4 | | -8.7 | | -0.6 |
| **Cohen's d** | | 0.28 | | 0.23 | | -0.36 | | -0.49 | | -0.30 | | -0.02 | | -0.34 | | -0.03 |
